# Inflammatory markers activation associated with vapor or smoke exposure in Wistar rats

**DOI:** 10.3389/fimmu.2025.1525166

**Published:** 2025-03-21

**Authors:** Ewelina Wawryk-Gawda, Michał K. Zarobkiewicz, Marta Wolanin-Stachyra, Violetta Opoka-Winiarska

**Affiliations:** ^1^ Department of Paediatric Pulmonology and Rheumatology, Medical University of Lublin, Lublin, Poland; ^2^ Department of Clinical Immunology, Medical University of Lublin, Lublin, Poland; ^3^ Department of Paediatrics, Pulmonology and Rheumatology, University Children's Hospital of Lublin, Lublin, Poland

**Keywords:** electronic cigarettes, vapor, immune, interleukins, smoke

## Abstract

Electronic cigarettes (e-cigarettes) were introduced two decades ago as a safer alternative to traditional cigarettes, aiming to assist in smoking cessation. However, the global use of e-cigarettes has surged, with the highest prevalence among adolescents and young adults. Despite their popularity, the safety of e-cigarettes remains controversial, with emerging evidence linking their use to various health risks, including cardiovascular issues, respiratory diseases, and a condition known as e-cigarette or vaping use-associated lung injury (EVALI). In this study, we investigated the inflammatory response in rats exposed to e-cigarette vapor compared to traditional cigarette smoke. We measured the serum concentrations of inflammatory markers such as IL-10, IFN-γ, IL-5, IL-2, TNF-α, GM-CS, IL-4, IL-9, IL-17F, IL-17A, IL-13, and IL-22 in the serum of rats subjected to 6 weeks of exposure. We assessed the activation of *Nf-κb*, *Stat3*, and *Socs3* genes and the expression of CXCL2 in lung tissues. Our results revealed a significant increase in proinflammatory cytokines, particularly in the vapor-exposed group. We did not observe any statistically significant difference in the activation levels of *Nf-κb*, *Stat3*, and *Socs3* between the groups of rats, but we noted the predictable correlations between IL-22 and IL-2, IL-6 and IL-2, IL-9 and IL-2, IL-6 and IL-9, IL-22 and IL-17F, IL-6 and IL-17F, IL-6 and IL-5, IL-2 and IL-17F, IL-13 and IL-4, and IL-5 and IL-4. In IHC staining, we observed a higher number of CLCX2-positive cells in the lung tissues in groups 2 and 3 compared to the control group. Interestingly, after a 2-week cessation period, inflammatory markers largely normalized, except for IL-17F and IL-13, which remained elevated in the cigarette smoke-exposed group. Our results suggest that while e-cigarette use may trigger a potent inflammatory response, the effects may be reversible upon cessation, albeit with some cytokines persisting longer in traditional cigarette users. Although the immune response has normalized, the increased tendency toward lung fibrosis may lead to permanent structural changes. Further research is needed to fully elucidate the clinical implications of these findings and assist in implementing legal regulations regarding the availability of e-cigarettes in the market.

## Clinical implications

**Table:** 

Clinical implications
Exposure to e-cigarettes causes a local inflammatory response in the lungs, which can lead to their damage and chronic respiratory disease. Patients with a predisposition to asthma or other respiratory conditions may experience exacerbated symptoms when exposed to e-cigarette vapor.
Prolonged e-cigarette use results in a chronic systemic inflammatory response resulting in organ changes, which can explain the development of diseases such as EVALI and cardiovascular symptoms, stroke, and myocardial infarction.
Evidence of inflammation in animals exposed to heated water vapor compared to smoke-exposed and control groups suggests that vaping may also induce an immune response.
The immune response to e-cigarettes is different from the response to traditional cigarettes, but it can also have serious health effects.
Education, including among young people, about the effects of vaping is very important for individuals but also for preventing population health effects.
Understanding the pathomechanism of diseases such as EVALI will enable the development of treatment recommendations for these conditions.

## Capsule summary

Vaping leads to inflammatory response activation, which can be the cause of lung injury; therefore, regulations regarding the availability of e-cigarettes in the market and anti-inflammatory drugs for e-cigarette-dependent disease treatment should be considered.

## 1 Introduction

Electronic cigarettes (e-cigarettes) were introduced nearly 20 years ago as a safer nicotine delivery system to aid in smoking cessation. However, in recent years, their global market has expanded significantly. The prevalence of e-cigarette use increased from 1.1% in 2019 to 2.4% in 2022. The increase in their availability and interest among young people is highly disturbing. The preference for electronic cigarettes over traditional ones is the highest among high school students (19.6% of smokers using e-cigarettes) (1, 2). In Germany, e-cigarettes with nicotine are used by approximately 41.6% of adolescents and 56.0% of young adults (3). The data concerning the safety of electronic cigarettes are inconclusive, and their use is also addictive, so the WHO does not recommend vaping as an aid in smoking cessation (2). However, evidence of harmful effects is needed to influence the decisions of people and relevant authorities.

E-cigarette aerosols contain a complex mixture of substances; therefore, the effect of e-cigarette substances on body cells is multidimensional. The liquid of e-cigarettes contains propylene glycol or vegetable glycerin, which acts as a vehicle for nicotine, together with other chemical components (flavors and other additives). Nicotine, a well-known toxic component of both tobacco smoke and e-cigarette aerosols, is not the only concern. Heating the liquid can release additional harmful substances, including metal and plastic particles from the device, which may exacerbate nicotine’s toxic effects (4, 5). The metals commonly used in e-cigarette coils include Kanthal, composed of iron, chromium, and aluminum, and nichrome, which consists of nickel and chromium. Additionally, metals like tin are employed in the joints. Exposure to these metals has serious health effects including neurotoxicity, cardiovascular disease, and respiratory disease such as lung cancer (4, 5). Kim et al. showed that e-cigarette aerosols of propylene glycol and vegetable glycerin may induce airway inflammation, increase the major mucin MUC5AC expression, and cause dysfunction of ion channels important for mucus hydration in primary human bronchial epithelial cells *in vitro*. Similar effects, such as increased metalloproteinase-9 activity and mucus concentration, have been observed in animal models (6).

Increasing evidence shows that the health consequences of using e-cigarettes can be serious. The available data have shown an increase of cardiovascular and death risks associated with the use of e-cigarettes. Moreover, several epidemiological observational studies have shown an association between vaping and wheezing, asthma, respiratory infection, and other respiratory tract diseases (1, 7, 8). An early onset of stroke is typical for e-cigarette users (9). The new disease, e-cigarette or vaping use-associated lung injury (EVALI), first recognized in March 2019 in the USA, is a toxic lung injury associated with using e-cigarettes, with early onset and violent course (10, 11). By February 2020, the U.S. Centers for Disease Control and Prevention (CDC) had documented over 2,800 cases and 68 deaths, though tracking the disease remains challenging (12). EVALI is a diagnosis of exclusion (13–16). The etiology remains unclear, and several causes are under investigation. Vitamin E acetate [a diluent in tetrahydrocannabinol (THC)-based cartridges] is the most recognized agent associated with EVALI. After removing much of the illicit THC from the market and through the COVID-19 pandemic, the number of patients with EVALI had decreased. Nevertheless, numerous patients still experience symptoms (17). The pathology of EVALI and other diseases associated with e-cigarette use is still poorly understood. Histological tests showed airway-centered chemical pneumonitis and a significant inflammatory reaction (17, 18). The results of studies performed on cell cultures, mice, and human subjects have revealed increased white blood cell counts and elevated inflammatory markers in bronchoalveolar lavage fluid and serum (13, 19). The management of EVALI involves cessation of e-cigarette use and supportive treatment. However, it is interesting that some patients have responded well to systemic corticosteroids, and this may indicate a role of inflammation in the pathogenesis of the disease (13–16, 20).

The primary aim of our study was to assess the inflammatory component of the response to e-cigarette exposure. This is an important research goal for understanding the pathogenesis of clinical complications of vaping and potential therapeutic approaches. The secondary objective was to compare the inflammatory response to e-cigarettes and conventional cigarettes. Studying and comparing the side effects of both methods of smoking has important practical health implications. The next secondary objective was to investigate the effect of smoking cessation. The available studies have indicated that smokers who quit between ages 25 and 34 have survival rates comparable to those who never smoked (1). By assessing inflammatory markers after 2 weeks of smoking or vaping cessation, we aimed to evaluate the potential for recovery. Our hypothesis was that inflammatory markers would normalize following this period.

To ensure a controlled experimental environment, we conducted our study using laboratory animals. Vapers and smokers use solutions of nicotine and additional ingredients that are supposed to be harmless to health. Therefore, we used real devices, liquid, and cigarettes in the experiment. The histological changes in lung structure were described in our previous publication (21). Our hypothesis postulates that lung injury may be caused by inflammation with activation of proinflammatory cytokines. Therefore, we selected cytokines and chemokines with well-established and explicit roles in inflammation, as their identification methods are relatively straightforward. In addition to blood tests, we examined inflammatory damage in the lung tissues of the animals. Specifically, we analyzed the expression of the *Nf-κb*, *Stat3*, and *Socs3* genes, along with the well-known chemoattractant CXCL2 in lung tissues. These markers were then correlated to validate the hypothesized inflammatory activation pathway.

## 2 Methods

### 2.1 Experiment

**Figure 1:**
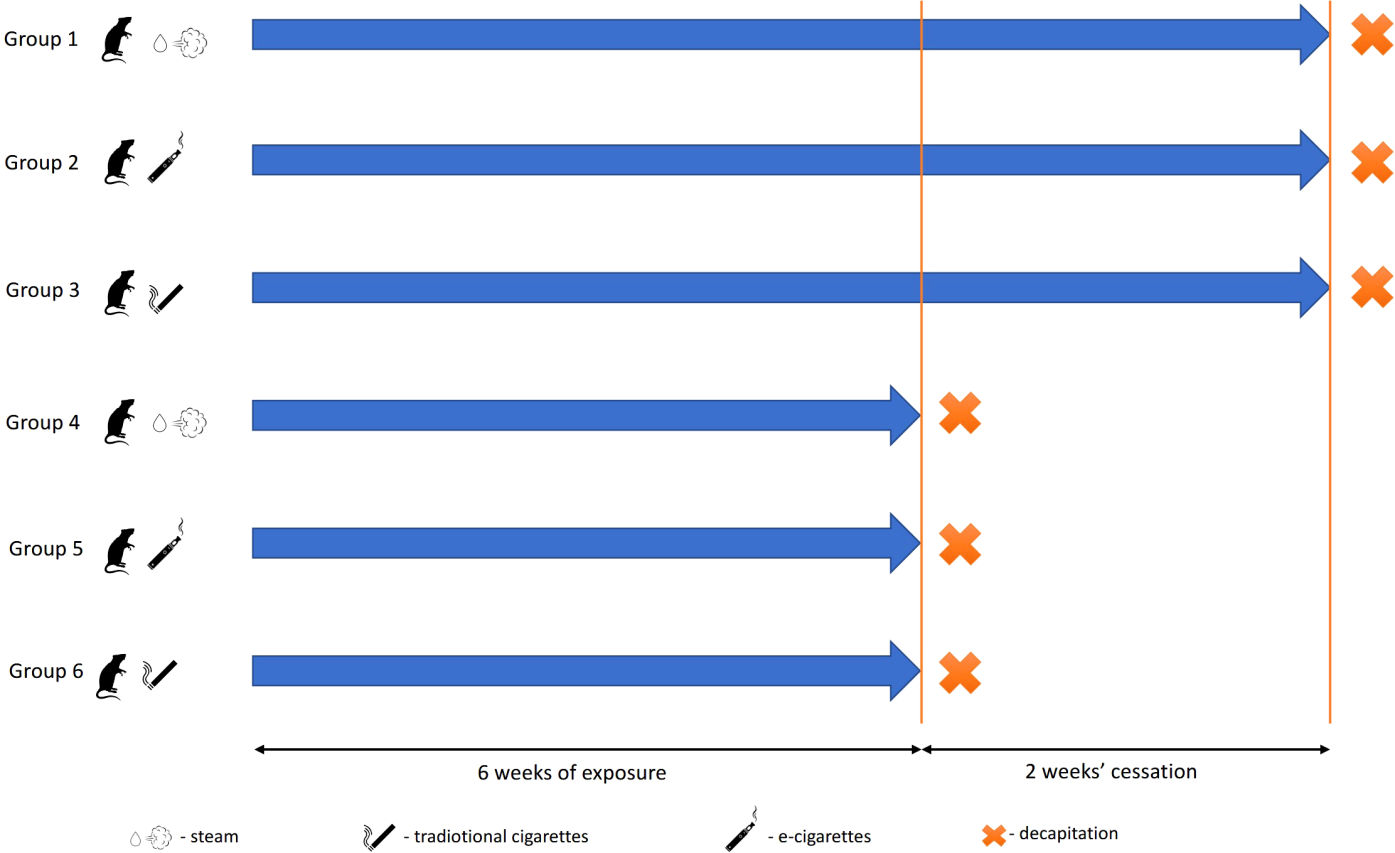
Experimental scheme. Groups decapitated after 24 h after the end of the experiment: 1, control; 2, exposed to vapor; and 3, exposed to smoke. Groups decapitated after a 2-week break from exposure: 4, control; 5, exposed to vapor; and 6, exposed to smoke.

The study was conducted using 30 male Wistar rats with an average initial body weight of 187.82 ± 12.56 g. The animals were divided into six groups, each housed in a uniform, controlled study environment (**Figure 1**).

#### 2.1.1 Exposure protocol

Rats of groups 2 and 5 were exposed to scent-free e-cigarette vapor. During each 30-min exposure session, the animals were placed in a 0.1-m³ PVC exposure chamber. A pump (0.18 kW; 1.4/1.6 A; 230 V; 50/60 Hz) was installed on one side of the chamber, while an e-cigarette continuously released aerosol from the opposite side, generating an airflow. The chamber, accommodating five animals at a time, was hermetically sealed, with only two openings for the pump and e-cigarette connection points. The animals were exposed to 0.6 ml/day of e-liquid containing 12 mg/ml of nicotine, propylene glycol, and water (e-liquid produced by Inawera Dot Com Sp. Z o.o. Sp. K., Turka, Poland). A single treatment cycle consisted of a 10-min puffing phase followed by a 20-min rest period. The voltage of the e-cigarette device was set at 5.5 V throughout the experiment. After exposure, animals were transferred to clean home cages. Exposure occurred once per day, 5 days per week, for six consecutive weeks. Rats of groups 3 and 6 were exposed to traditional cigarette smoke equivalent to the total nicotine dose received by group 2. Over the entire study period, each animal received a cumulative nicotine exposure of 210 mg. Groups 1 and 4 (control groups) underwent the same inhalation-related procedures as groups 2 and 3 but without nicotine exposure to account for stress effects from inhalation.

#### 2.1.2 Sample collection and analysis

Animals of groups 1–3 were decapitated without anesthesia 24 h after the last exposure. Groups 5 and 6 underwent 6 weeks of exposure, followed by a 2-week recovery period without exposure before euthanasia. Blood and lung samples were collected immediately postmortem. Lung tissues were fixed in 10% buffered formalin, embedded in paraffin blocks, and sectioned into 5-μm-thick slices for histological examination. For RT-PCR, lung samples were rapidly frozen in liquid nitrogen and stored at −70°C. Cotinine levels in urine were measured using an ELISA kit (#KA1416, Abnova, Taipei, Taiwan; **Supplementary Figure S1**).

#### 2.1.3 Ethical considerations and study approval

Only healthy, certified Wistar rats from the Center for Experimental Medicine in Lublin were used. The animals were continuously monitored by a veterinarian and remained in good physical and behavioral condition throughout the experiment. The study adhered to Directive 2010/63/EU of the European Parliament on the protection of animals used for scientific purposes. The protocol received formal approval (136/2018) from the Local Ethics Committee for Animal Experiments at the University of Life Sciences in Lublin. Sample size estimation was performed using Statistica software [power analysis for one-way analysis of variance (ANOVA)] with the following parameters: number of groups = 3; alpha level = 0.05; power = 0.8; and group means = calculated based on preliminary research and data in the literature.

### 2.2 Laboratory tests

#### 2.2.1 The concentrations of cytokines in rats’ serum

LEGENDplex™ Rat Th Cytokine Panel (13-plex) manufactured by BioLegend (San Diego, USA) was used to assess the concentrations of cytokines in rats’ serum. The LEGENDplex™ Rat Th Cytokine Panel is a bead-based multiplex assay panel, using fluorescence-encoded beads suitable for use on various flow cytometers. This panel allows simultaneous quantification of 13 rat cytokines, namely, IL-10, IFN-γ, IL-5, IL-2, tumor necrosis factor-α (TNF-α), granulocyte–macrophage colony-stimulating factor (GM-CSF), IL-4, IL-17F, IL-9, IL-17A, IL-13, and IL-22. This assay panel provides higher detection sensitivity and broader dynamic range than traditional ELISA methods. The panel has been validated for use on cell culture supernatant, serum, and plasma samples. The tests were performed according to the manufacturers’ protocols, and the results were presented in pg/ml. Three samples from each rat were analyzed with CytoFlex LX (Beckman Coulter, Warsaw, Poland).

#### 2.2.2 Real-time qPCR of lung tissues

Frozen lung samples were cut into small parts with a scalpel, and total RNA was immediately isolated with ExtractMe Total RNA Kit (Blirt, Gdańsk, Poland). Then, cDNA was obtained using the TranScriba Kit (A&A Biotechnology, Gdańsk, Poland). Finally, qPCR reactions were prepared with the qPCR-HS Mix SYBR master mix (A&A Biotechnology) with the primers listed in **Table 1**. Reactions were run on ViiA 7 or ABI7300 (Applied Biosystems, Carlsbad, CA, USA). *Actb* and *Gapdh* were used as housekeeping genes. The 2−ΔCT formula was used to calculate the relative expression (22).

**Table 1:** Primer sequences for RT-PCR.

Gene	Forward primer (5′−3′)	Reverse primer (5′−3′)
*Nf-κb*	*TTCAACATGGCAGACGACGA*	*AGGTATGGGCCATCTGTTGAC*
*Stat3*	*GTACAATCCCGCTCGGTGT*	*TTTGTTGGCGGGTCTGAAGT*
*Socs3*	*CCCCGCTTTGACTGTGTACT*	*AAAGGAAGGTTCCGTCGGTG*
*Gapdh*	*GAAGGGCTCATGACCACAGT*	*TATTCGAGAGAAGGGAGGGC*
*Actb*	*CCCGCGAGTACAACCTTCTT*	*TCATCCATGGCGAACTGGTG*

#### 2.2.3 IHC staining

Immunohistochemical staining of lung tissues was performed with the use of antibodies directed against CXCL2 (Rabbit pAb Abclonal, A12639-20), diluted 1:500 in SignalStain^®^ Antibody Diluent. Exposure of the antigenic sites was performed thermally by incubation in an unmasking solution with pH = 6, in a microwave oven at 800 W, for three cycles lasting 5 min each. In order to inhibit endogenous peroxidase activity, slides were incubated in 0.3% Perhydrol (H_2_O_2_) in methanol for 30 min. The samples were incubated in Animal-Free Blocking Solution (Danvers, United States: Cell Signaling Technology, Inc.) for 1 h to block the non-specific bindings. The material was incubated at 25°C for 1 h in a diluted primary antibody. As a secondary antibody, SignalStain ^®^ Boost IHC Detection Reagent (HRP, Rabbit) was used. The diaminobenzidine solution (DAB, SignalStain ^®^ DAB Substrate Kit) and hematoxylin coloration were used to visualize the reaction (5 min). Negative controls were prepared in a similar manner, and only the specific primary antibody was omitted. The material was evaluated with a light microscope using lenses ×10 and ×40. The cells were counted in 100 fields of view using a microscope with digital camera Olympus BX41 under ×400 lens using cellSens software (Olympus Corporation). The percentage of CXCL2-positive cells was evaluated by two independent scientists.

### 2.3 Statistical analysis

Means ± standard deviation, standard error, and 95% confidence interval of the tested cytokines were calculated, and statistical analysis was performed using Statistica Software (StatSoft Polska 14.1.04 Cloud Software Group Inc.) and Stata BE 18.5 (StataCorp LLC, Texas, USA). The Shapiro–Wilk test was used to assess whether the distribution of the variables was normal. The main concentration of the cytokines and the activation levels of the tested genes of each experimental group were compared to the control, and the level of significance was tested using the one-way ANOVA test followed by Tukey’s *post-hoc* test. A correlation between the tested cytokines was made using Spearman’s correlation test. The distribution of the IHC staining results was not normal; therefore, they were analyzed with the non-parametric Kruskal–Wallis test. Statistical significance was set at *p*-value <0.05. The power of the test and the sample size were adequate (>0.7 in the power test). The graphs were created using PRISM V9 software (GraphPad, San Diego, CA, USA).

## 3 Results

### 3.1 The concentrations of cytokines in rats’ serum

**Figure 2:**
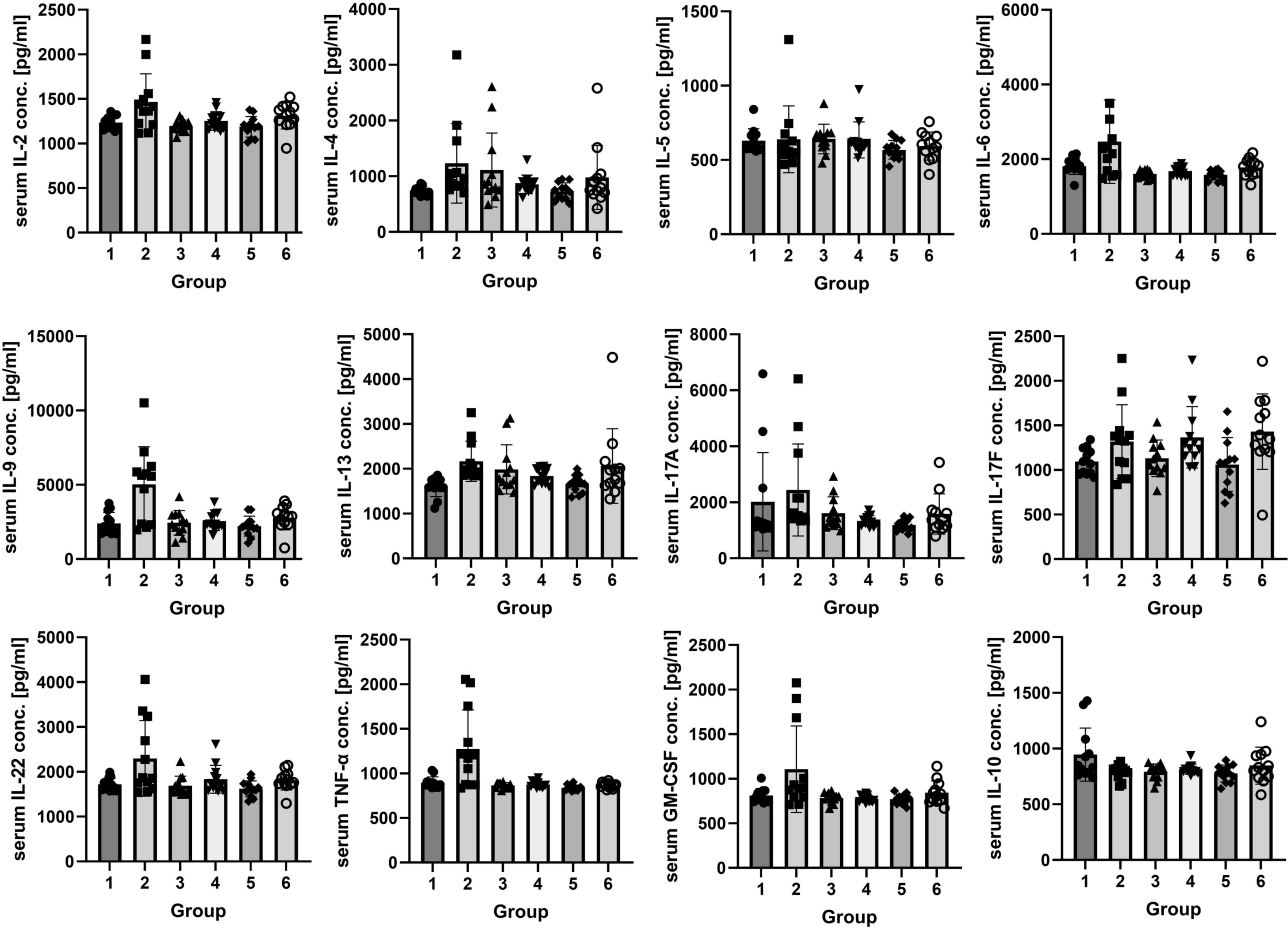
The concentrations of IL-2, IL-4, IL-5, IL-6, IL-9, IL-13, IL-17A, IL-17F, IL-22, IL-10, GM-CSF, and TNF-*α* (pg/ml) in rats’ serum. Twenty-four hours after the end of the experiment, the concentrations of IL-2, IL-6, IL-9, IL-22, GM-CSF, and TNF-*α* were higher in group 2 than in the control group and group 3 (*p* < 0.05). The concentration of IL-4 was not statistically significantly higher in the vapor-exposed group (1,231.17 ± 712.55 pg/ml) than in the control group (737.28 ± 72.97 pg/ml) and in the smoke-exposed group (1,112.19 ± 663.78 pg/ml). The concentration of IL-13 was higher in the second group than in the control group. The concentration of IL-17A was the highest in group 2 (2,442.14 ± 1,644.20 pg/ml) and the lowest in group 3 (1,608.31 ± 586.67 pg/ml), and the concentration of IL-17F was the highest in group 2 (1,318.44 ± 414.37 pg/ml), but the differences were not statistically significant. The concentration of IL-10 was lower in groups 2 and 3 than in the control group. The concentrations of IL-5 were similar in every group. After a 2-week break, IL-17F was lower in group 5 than in the control and the smoke-exposed group (*p* < 0.05); the concentrations of IL-2, IL-4, IL-6, IL-9, IL-13, IL-17A, and GM-CSF were higher in group 6 than in group 5 and the control group, but the differences were not statistically significant. IL-22 and TNF-*α* concentrations were on a similar level in every group. IL-10 was insignificantly the lowest in group 5. Groups decapitated after 24 h after the end of the experiment: 1, control; 2, exposed to vapor; and 3, exposed to smoke. Groups decapitated after a 2-week break from exposure: 4, control; 5, exposed to vapor; and 6, exposed to smoke.

The analysis of rats’ serum showed an increase of inflammatory marker concentrations in the groups exposed to vapor and smoke compared to the control. The highest increase of proinflammatory markers (IL-2, IL-6, IL-9, IL-22, GM-CSF, and TNF-*α*) was observed in the group exposed to vapor (**Figure 2**). IL-5 and INF-*γ* were on a similar level in every group (*p* > 0.05, not shown in the figure). Anti-inflammatory IL-10 was decreased in the groups exposed to vapor and smoke (**Supplementary Tables S1**, **S2**).

The results showed a clear trend toward higher values of interleukins (IL-2, IL-4, IL-6, IL-9, IL-13, IL-17A, and GM-CSF) in the smoke-exposed group than in the vapor-exposed and control groups, but the differences were not statistically significant. IL-22 and TNF-*α* concentrations were on a similar level in every group. IL-10 showed a trend toward the lowest values in the vapor-exposed group, but the differences were not statistically significant (*p* > 0.05, **Supplementary Tables S1**, **S2**).

After a 2-week break from vapor or smoke exposure, the concentrations of the cytokines mostly normalized. Only IL-17F was higher in the group exposed to vapor than in the smoke-exposed group (*p* < 0.05) and insignificantly higher than in the control group.

### 3.2 Real-time qPCR of lung tissues

We did not observe any significant difference in the expression levels of the *Nf-κb*, *Stat3*, and *Socs3* genes between the groups of rats (**Supplementary Figure S1**).

### 3.3 Correlation between the tested proteins and genes

**Figure 3:**
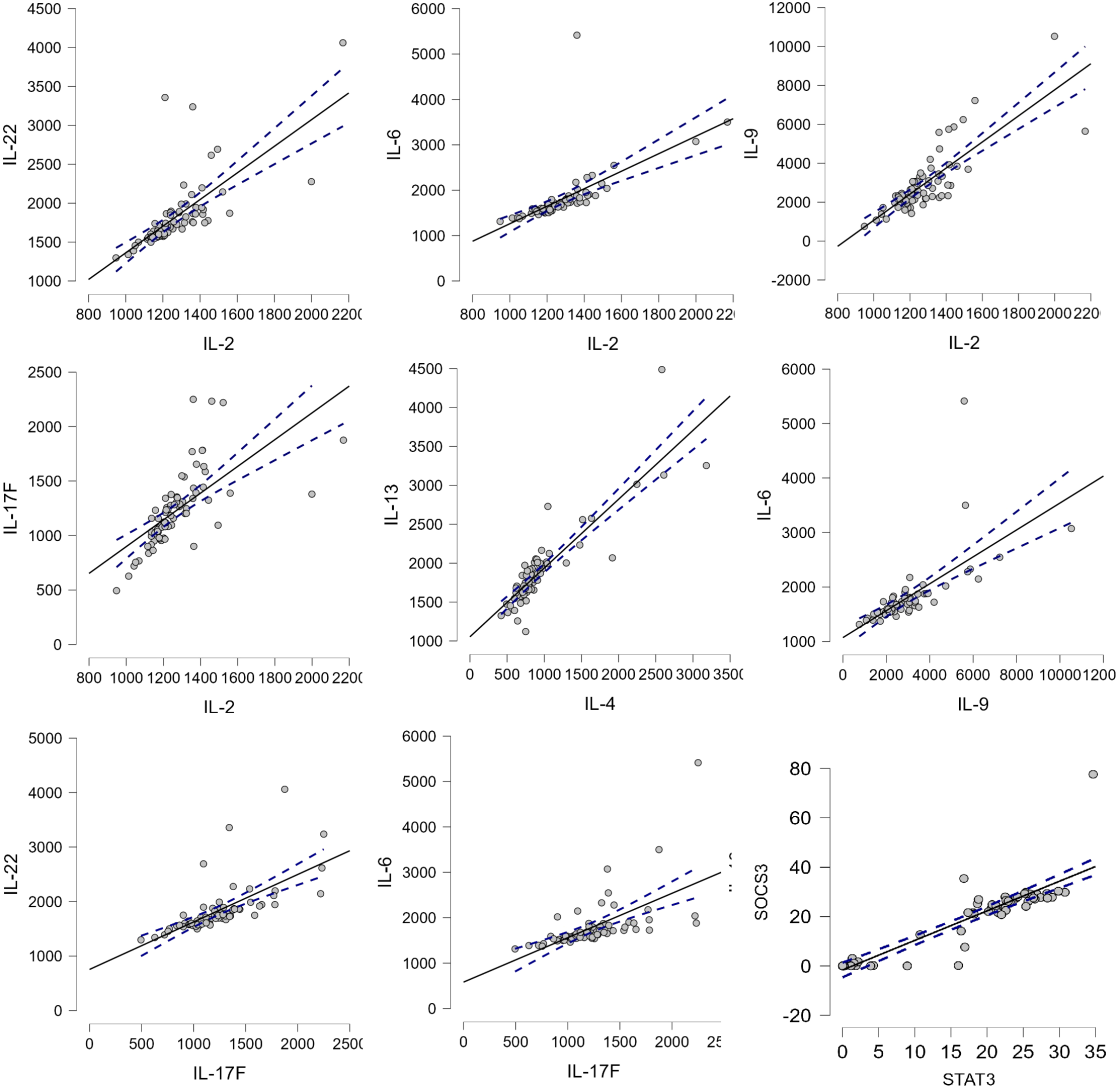
The correlation between cytokines. *p*-value <0.05, Spearman’s rho: IL-22–IL-2, 0.867; IL-6–IL-2, 0.904; IL-9–IL-2, 0.779; IL-17F–IL-2, 0.843; IL-13–IL-4, 0.882; IL-6–IL-9, 0.838; IL-22–IL-17F, 0.842; IL-6–IL-17F, 0.807; and SOCS3–STAT3, 0.896.

The study confirmed the correlations between proinflammatory cytokines in Spearman’s correlations test: IL-22 and IL-2, IL-6 and IL-2, IL-9 and IL-2, IL-6 and IL-9, IL-22 and IL-17F, IL-6 and IL-17F, and IL-6 and IL-5. Moreover, cytokines of the Th2 pathway were also correlated: IL-2 and IL-17F, IL-13 and IL-4, and IL-5 and IL-4 (**Figure 3**).

### 3.4 IHC staining with the CXCL2 antibody in lung tissues

**Figure 4:**
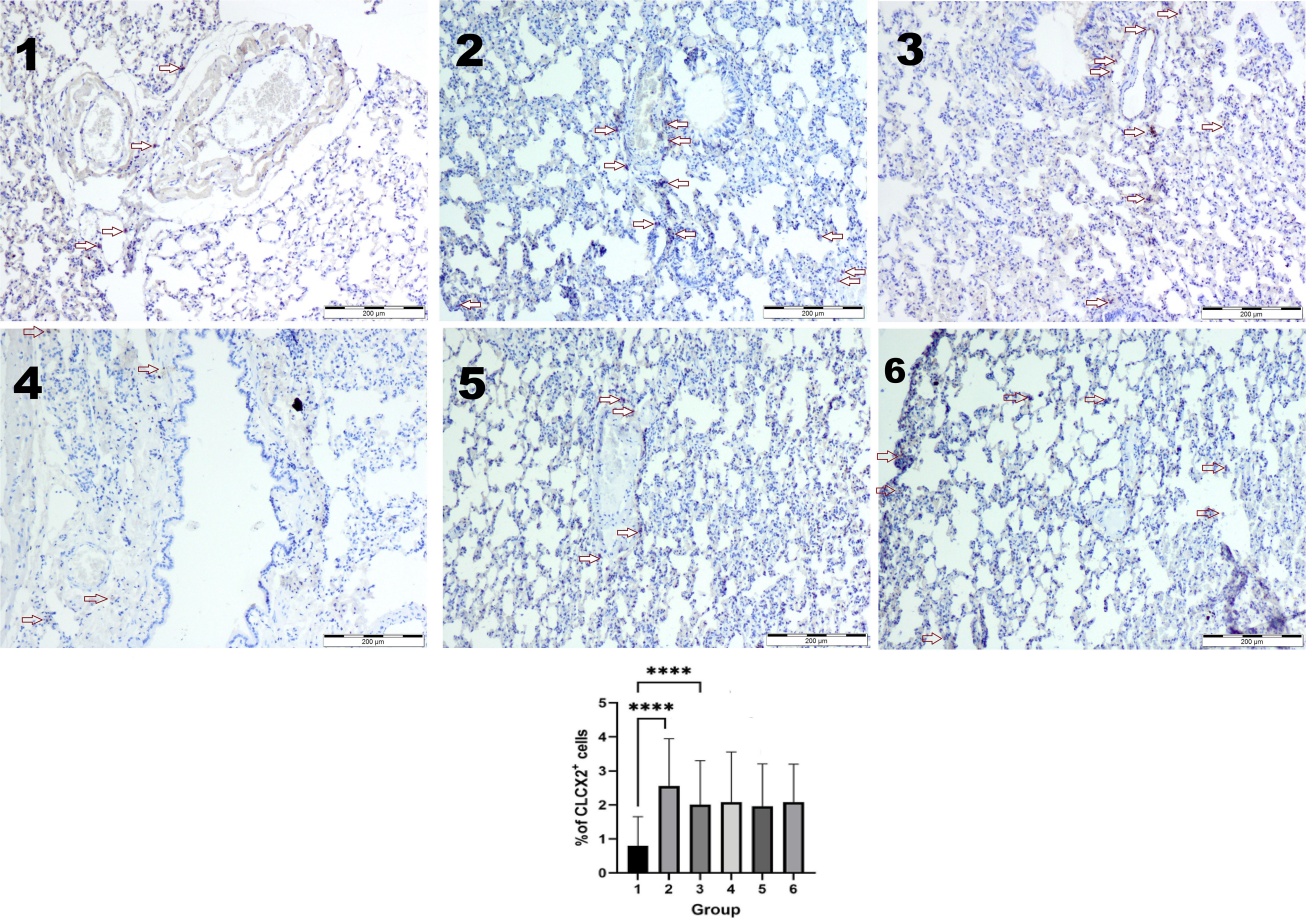
Immunohistochemical reaction with the CXCL2 antibody. The higher number of CLCX2-positive cells was observed in groups 2 and 3 compared to the control (1) group (p < 0.05 in the Kruskal–Wallis test ****). Two weeks after the end of exposure, the number of CXCL2-positive cells was similar in every group (4–6). IHC staining. Original magnification, ×100.

The higher number of CLCX2-positive cells in the lung tissues was observed in the groups exposed to vapor or smoke compared to the control group (*p* < 0.05 in the Kruskal–Wallis test). Two weeks after the end of exposure, the number of CXCL2-positive cells was similar in every group (**Figure 4**).

## 4 Discussion

Our study demonstrates a significant inflammatory response in both blood and lung tissues following exposure to cigarette smoke and e-cigarette vapor. We observed notable differences in inflammatory marker concentrations in the serum, particularly elevated levels of IL-2, IL-6, IL-9, IL-22, GM-CSF, and TNF-α in vapor-exposed rats compared to those exposed to smoke or in the control group. These findings suggest a differential immune response that may have implications for understanding the varied impacts of traditional smoking and vaping on pulmonary and systemic inflammation.

Our results align with those of Dai et al., who reported increased TNF-α levels in bronchoalveolar lavage fluid (BALF) and elevated IL-6 in both BALF and serum in e-cigarette-exposed mice compared to controls (19). Wang et al. also observed a significant increase in proinflammatory cytokines in BALF from mice exposed to e-cigarette aerosols containing propylene glycol and nicotine compared with air‐exposed control mice (23).

However, unlike our findings, Dai et al. noted slightly lower cytokine levels in the e-cigarette group compared to traditional cigarette exposure. This discrepancy may stem from the shorter exposure duration in their study (3 weeks vs. 6 weeks in ours), suggesting that prolonged vapor exposure may result in a more pronounced consequence (19). Similarly, Alzoubi et al. observed increased TNF-α levels in the lung tissues over 1, 2, and 4 weeks of exposure; no significant IL-6 changes were detected in the lungs or BALF (23). Notably, Sussan et al. found lower IL-6 concentrations in BALF and lung homogenate after 2 weeks of vapor exposure, which contrasts with the findings from our study, further emphasizing the importance of exposure duration (24). Belkin et al. conducted a human study that found a significant increase in IL-6, IL-2, and TNF-α levels in e-cigarette users shortly (120 min) after device usage, which is aligned with our results of elevated inflammatory markers in the serum (18). Contrary to our results, in this study, we failed to detect significant changes in the concentrations of IL-4, GM-CSF, INF, and IL-10 for any of the groups (18), while in the study of Lim et al., the concentrations of IL-4, IL-13, and IL-5 were higher in the BALF of mice exposed to e-cigarettes by 10 weeks (25, 26).

The involvement of cytokines, chemokines, and transcription factors in the pathogenesis of lung diseases is currently being extensively studied. Immunological abnormalities have been proven to lead to acute lung injury (ALI), acute respiratory distress syndrome (ARDS), asthma, chronic obstructive pulmonary disease (COPD), EVALI, and idiopathic lung fibrosis (IPF) (27). The role of cytokines, especially IL-6, TNF-α, and GM-CSF, in mediating inflammatory responses is well-documented. IL-6, in particular, is involved in acute-phase reactions and is a prognostic biomarker in various acute organ injuries. IL-6 is promptly produced and contributes to host defense when infections or tissue injuries occur. On the other hand, excessive IL-6 production has been linked to the development of acute and life-threatening complications such as systemic inflammatory response syndrome (SIRS) and cytokine-release syndrome. Moreover, IL-6 inhibits regulatory T (Treg) cell differentiation, which plays a role in immune homeostasis and preventing inflammation. Affecting the Th17/Treg balance, IL-6 takes part in the pathogenesis of chronic autoimmune and inflammatory disorders such as autoimmune hemolytic anemia, rheumatoid arthritis, systemic sclerosis, inflammatory bowel disease, and asthma (28).

Our study also identified increased IL-2, IL-4, IL-9, and IL-13 concentrations, which are associated with allergic inflammation and airway remodeling. IL-9 activates mast cells that produce IL-13, which, in turn, affects lung epithelial cells. IL-13 is primarily produced by Th2 lymphocytes but also by eosinophils and NK cells and is a key mediator of asthma and allergy-related inflammation. We found that IL-13 levels were significantly higher following e-cigarette exposure compared to traditional smoking. After a 2-week cessation period, IL-13 levels in the vapor-exposed group decreased, while they remained unchanged in smoke-exposed rats. These findings suggest that sustained IL-13 elevation could contribute to chronic airway inflammation and increased susceptibility to respiratory diseases (29–32). IL-13 with IL-33 synergistically stimulates the polarization of M2 macrophages that play an important role during the late pulmonary fibrosis phase (27, 33). The positive correlation between IL-4 and IL-13 and between IL-2 and IL-17F noted in our study suggests that the Th2 inflammatory pathway is responsible for histological changes observed in our previous study. In their study, Scott et al. highlighted the dominant role of IL-4 and IL-13 in airway remodeling and a decrease of lung function. Vascular remodeling accelerated by IL-4 and IL-13 is a structural change impacting asthma and COPD pathophysiology, leading to decreased lung capacity and airflow (34).

IL-22, which plays a critical role in epithelial barrier function and lung tissue repair, was notably elevated in vapor-exposed groups (35). However, studies suggest that nicotine suppresses IL-22 receptor expression, potentially impairing lung repair and contributing to chronic lung damage (36). The correlation between IL-22 and IL-17F in our study suggests a role in neutrophil and eosinophil recruitment, which could lead to tissue destruction and fibrosis. This aligns with previous research indicating that IL-22 and IL-17 play homeostatic roles in the lung and may contribute to chronic inflammation and fibrotic remodeling (21, 35, 37).

Recent research has shown the transition of macrophages from a resting state to the M1 phenotype primarily through the glycolytic pathway that leads to the production of significant amounts of nitric oxide (NO) and reactive oxygen species (ROS). These processes amplify the inflammatory response by activating intracellular pathways involving proteins such as nuclear factor-kappa B (NF-κB). The antioxidative mechanisms such as peroxide dismutase, catalase, and glutathione are activated to protect cells against ROS. Finally, the transcription factor signal transducer and activator of transcription (STAT) pathway is activated, and the polarization of macrophages results in the formation of the M2 phenotype (27). In our study, we did not observe any significant differences in *Nf-κb*, *Stat3*, and *Socs3* between the groups. However, the correlation between *Stat3* and *Socs3* was significant, which may suggest the important role of the JAK–STAT signaling pathway in lung injury caused by vapor or smoke exposure (38). The data indicate that STAT3 phosphorylation is crucial for the polarization of macrophages into the M2 phenotype and the secretion of IL-4 and IL-10. The high expression of STAT3 has been noted in lung biopsies of animals and patients with IPF. SOCS3 is responsible for the regeneration of lung tissues in response to different stimuli. Wu et al. reported increased SOCS3 expression along with activation of the STAT3/NF-κB pathway in obese mice. This aligns with the established understanding of chronic inflammation associated with obesity, where SOCS3 acts as a negative feedback regulator to modulate inflammatory responses. Furthermore, pharmaceutical stimulation of SOCS3 expression in non-obese mice leads to inhibition of the JAK2–STAT3 pathway, reduction of JAK2–STAT3/NF-κB signaling, and alleviation of lung injury. In contrast, SOCS3 knockdown in obese mice exacerbated lung damage (38). The anti-inflammatory properties of SOCS3 have also been proven in the study by Kim et al., where SOCS3 was an important mediator for macrophage modulation after mesenchymal stem cell transplantation (39). Moreover, the regulatory role of SOCS3 in COPD appears to be overshadowed by other factors, deviating from the patterns observed in animal models. According to Tine et al., measuring SOCS3 expression in BAL macrophage-derived extracellular vesicles could serve as an indicator of inflammation severity and potential COPD progression. Additionally, miRNA-mediated downregulation of SOCS3 in smokers without COPD may increase their susceptibility to developing cancer (40).

We previously reported structural lung damage in rats exposed to smoke or vapor, characterized by alveolar collapse, thickened alveolar septa, and mucus accumulation in bronchioles. The present study expands on these findings by demonstrating increased chemokine concentrations in serum and lung tissue, which likely contribute to inflammatory cell recruitment and tissue permeability. Elevated levels of CXCL2, a macrophage inflammatory protein, may explain the persistent inflammatory response and airway smooth muscle migration observed in our study. CXCL2 has been implicated in both protective and pathological immune responses, influencing neutrophil recruitment and airway remodeling. Proinflammatory cytokines such as IL-2, IL-6, IL-9, and TNF-α together with CXCL2 [macrophage inflammatory protein 2 (MIP-2)] secreted by macrophages, leukocytes, and cytokine-activated endothelial and epithelial cells play an important role in inflammatory cell recruitment (21). If neutrophils and macrophages infiltrate the tissue, they release proteolytic enzymes. Neutrophils and their products increase endothelial and epithelial permeability, in part due to apoptotic and necrotic cell death. This process is driven by the activation of the protein kinase C (PKC)/NADPH pathway, which is triggered downstream of CXCL2 binding to CXCR2. In a study conducted by Wang et al., extracellular vesicles containing CXCL2 were found to activate the CXCR2/PKC/NOX4 pathway, contributing to tissue injury (26). Zhou et al. observed that also in influenza and COVID-19 infections (41). In our previous study, elastolysis was observed; in both experimental groups, elastic fibers were disrupted, sparse, irregular, and thickened. Moreover, in both groups, we previously described more numerous α-SMA-positive myofibroblasts and blood vessels that were the typical symptoms of initial fibrosis, the long-term consequence of chronic inflammation in lung tissue caused by fibroblast proliferation and excessive collagen deposition (21). In our current study, we observed an increase of CXCL2 in the smoke- or vapor-exposed group of rats, which may be responsible for the persistence of active inflammatory response and the migration of airway smooth muscle cells taking part in remodeling (33, 42, 43).

The protective anti-inflammatory role played by IL-10 is vital to protect from excessive inflammation and related tissue damage; in our study, the concentration of IL-10 was decreased in the vapor- and smoke-exposed groups and stayed low after 2 weeks in the vapor group. This finding aligns with previous research by Alzoubi et al., who reported significant reductions in IL-10 levels in lung tissue after 1 week of exposure, though no corresponding changes were detected in BALF (44). The sustained slightly low concentration of IL-10 in the vapor group, even after cessation of exposure, could imply a prolonged impairment of the lung’s anti-inflammatory defenses, which may contribute to long-term respiratory complications.

Jacobs et al. reported that the addition of cigarette smoke extract (CSE) to B cells resulted in a reduced capacity to produce IL-10. This reduction may be mechanistically explained by the decreased levels of key transcription factors involved in IL-10 production, namely, IRF4 and HIF-1α, upon CSE exposure. IRF4 plays a crucial role in B-cell activation and differentiation, while HIF-1α is involved in cellular responses to hypoxia and inflammation. Both transcription factors are essential for IL-10 induction, and their suppression due to cigarette smoke exposure likely contributes to the observed impairment in Breg function (45, 46).

The observed imbalance in immune response caused by vapor or smoke exposure may be a factor in the development of diseases such as EVALI, stroke, and myocardial infarction (9, 13–17, 29). E-cigarette users had an earlier onset of stroke in comparison with traditional smokers in the study of Patel et al. (median age: 48 vs. 59 years; *p* < 0.0001). The authors found the acute harmful effects of e-cigarettes on vascular function, including high blood pressure and heart rate, increased arterial stiffness, endothelial dysfunction, and increased cerebrovascular oxidative stress (9). The data explaining EVALI pathogenesis have shown that the most common EVALI patients were healthy white male adolescents or young adults who used illicit THC-containing e-cigarettes. The primary histopathological finding was diffuse alveolar damage, often accompanied by bilateral ground-glass opacities observed in chest radiographs or CT scans. Additionally, bronchoalveolar lavage samples showed increased presence of macrophages or neutrophils, as well as foamy macrophages, M1 phenotype, and high cytokine profile (20). Conversely, e-cigarette users who do not develop EVALI exhibit reduced inflammatory cytokine production and show characteristics linked to reparative (M2) phenotype macrophages. These findings suggest that e-cigarette users who develop EVALI experience macrophage-specific alterations (17).

We have demonstrated the presence of substantial inflammation following exposure to e-cigarette use. Importantly, the inflammatory process is not limited to the lungs and affects the organism in general, as evidenced by proinflammatory cytokines present in the blood. This may explain the cardiovascular changes in e-cigarette smokers. This can be especially dangerous for people who already suffer from inflammatory diseases, such as asthma. The importance of environmental factors in the pathogenesis of autoinflammatory and autoimmune diseases is widely acknowledged. Among these, exposure to e-cigarettes should be considered. In 2022, the FDA gained regulatory control over synthetic nicotine e-cigarettes, effectively closing a loophole previously used by e-cigarette manufacturers to bypass FDA oversight. Despite this, many e-cigarette products remain easily accessible and largely unregulated, including those containing cannabinoids and other substances outside the scope of the FDA authority. This lack of regulation may allow untested compounds to be included in e-liquids, which could pose inhalation risks and contribute to future EVALI outbreaks (47). We believe that our study expands the information about the significant inflammatory response to e-cigarette exposure. This is important also for general health information for people and the decisions of relevant authorities in spreading access to stimulants (27).

Our study has certain limitations that should be acknowledged. Our study was conducted on a single strain of laboratory animals for a limited period of 6 weeks. Only male rats were used in this study to maintain homogeneity within the experimental groups and to eliminate potential hormonal influences that could introduce variability in inflammatory responses. The fluctuations in estrogen and progesterone levels in female rats could affect immune and inflammatory pathways, making it more challenging to isolate the specific effects of e-cigarette exposure. Future studies should include both male and female subjects to assess potential sex-based differences in response to e-cigarette exposure. Only one type of e-cigarettes was used. The inflammatory markers were measured in the serum and lung tissues of rats, so it is difficult to make a comparison with studies using BALF. In accordance with current directives, the number of animal groups was as small as possible but sufficient for the conclusions. Further studies should include intersex comparison using different types of nicotine delivery systems and different tested materials.

## 5 Conclusion

In conclusion, our study adds to the growing body of evidence that vaping can lead to significant alterations in cytokine profiles, with potential long-term implications for health. The differences in inflammatory marker levels between vapor and smoke exposure underscore the need for further research to fully understand the health risks associated with vaping, especially in comparison to traditional cigarette smoking. In the case of a short-term health history, it leads to recovery. Exposure to e-cigarettes is usually chronic. Chronic inflammation can result in tissue, organ, and then systemic damage, not limited to the respiratory system that we showed in our previous study (21, 48).

Future studies should explore the long-term effects of these exposure methods, particularly in the context of chronic inflammatory diseases and the potential for recovery post-exposure.

Human body development continues into the early 20s, and adolescents who vape are at risk for stunting or altering the development of their lungs, heart, and other organs, potentially preventing them from reaching their full functional potential. Vaping can also modulate the inflammatory and immune systems, increasing the risk of the development of disease over time. Thus, there is an urgent need for strict new regulations on e-cigarette access, sales, and marketing (8, 10).

## Data Availability

The original contributions presented in the study are included in the article/**Supplementary Material**. Further inquiries can be directed to the corresponding author.

## Supplementary material

Supplementary Figure 1 The cotinine concentration [ng/ml] in urine or rats after vapor (group 2), smoke (group 3) or saline (group 1) exposure in different weeks of experiment. Tested in urine samples using ELISA kit.

Supplementary Figure 2 Real-time PCR. The difference in the activation levels of the genes: *Nf-κb, Stat3, Socs3* between groups or rats weren’t statistically significant.
