# Supplementary figures and images for: Inflammatory markers activation associated with vapor or smoke exposure in Wistar rats
Source: Front Immunol. 2025 Mar 21;16:1525166. doi: 10.3389/fimmu.2025.1525166 (PMC11968385; doi:10.3389/fimmu.2025.1525166)

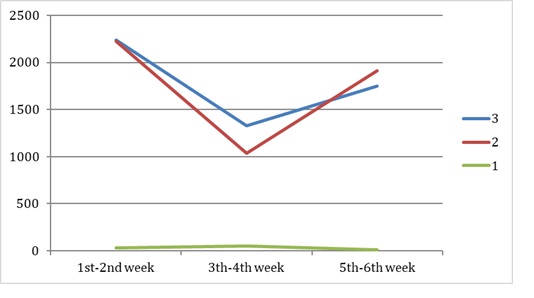

Supplement: Supplementary Figure 1 — The cotinine concentration [ng/ml] in urine or rats after vapor (group 2), smoke (group 3) or saline (group 1) exposure in different weeks of experiment. Tested in urine samples using ELISA kit. [file Image1.jpeg]

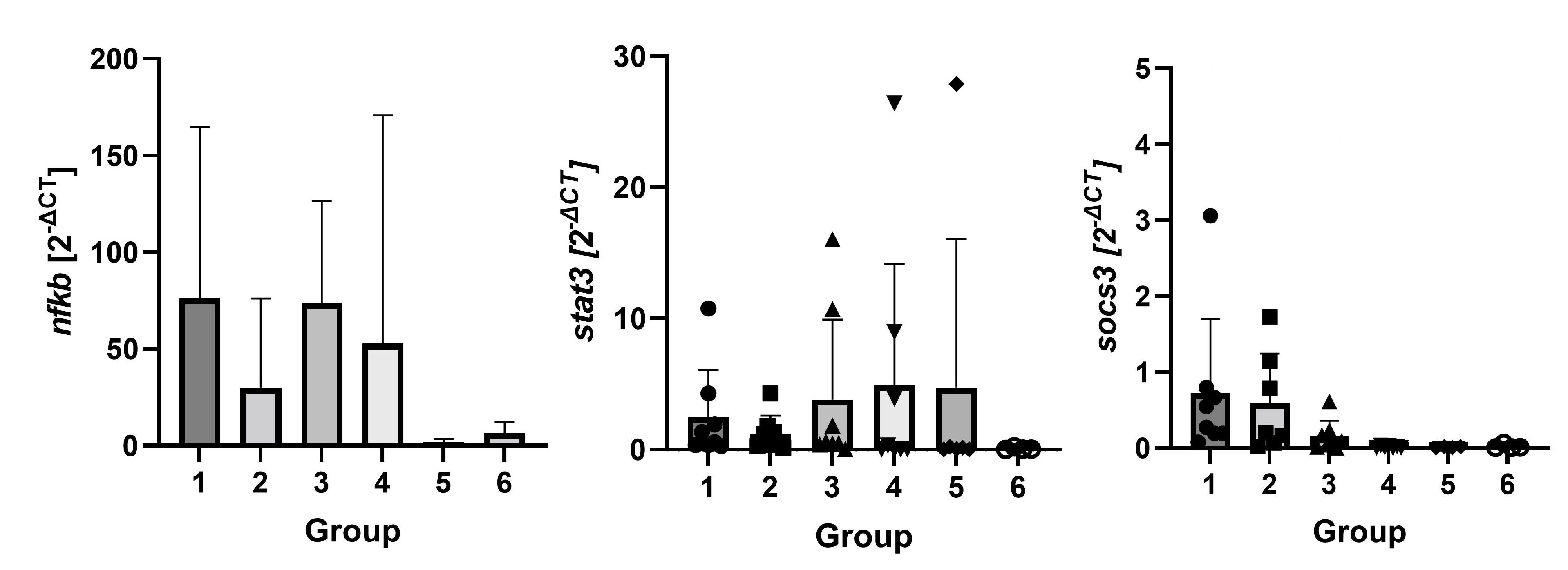

Supplement: Supplementary Figure 2 — Real-time PCR. The difference in the activation levels of the genes: Nf-κb, Stat3, Socs3 between groups or rats weren’t statistically significant. [file Image2.jpeg]
