# Supplementary material for: Inflammatory markers activation associated with vapor or smoke exposure in Wistar rats
Source: Front Immunol. 2025 Mar 21;16:1525166. doi: 10.3389/fimmu.2025.1525166 (PMC11968385; doi:10.3389/fimmu.2025.1525166)
Supplement: Supplementary file 3 [file Table1.docx]

## **Supplementary material**

Table I. p value in Tukey’s test. Statistical analysis of cytokines concentration.

| cytokine | P value in post-hoc Tukey’s test of One Way Analysis of Variance (ANOVA). | | | | | |
| --- | --- | --- | --- | --- | --- | --- |
|  | 1vs2 | 1vs3 | 2vs3 | 4vs5 | 4vs6 | 5vs6 |
| IL-2 | 0.02 | 0.99 | <0.01 | 0.93 | 0.95 | 0.46 |
| IL-4 | 0.12 | 0.38 | 0.99 | 0.99 | 0.99 | 0.79 |
| IL-5 | 0.99 | 0.99 | 1.0 | 0.78 | 0.96 | 0.99 |
| IL-6 | <0.01 | 1.00 | <0.01 | 1.00 | 0.99 | 0.90 |
| IL-9 | <0.01 | 1.00 | <0.01 | 0.99 | 0.99 | 0.87 |
| IL-10 | 0.04 | 0.04 | 1.00 | 0.98 | 0.99 | 0.77 |
| IL-13 | 0.04 | 0.32 | 0.93 | 0.93 | 0.85 | 0.30 |
| IL-17A | 0.92 | 0.93 | 0.39 | 1.00 | 0.99 | 0.94 |
| IL-17F | 0.54 | 1.00 | 0.71 | 0.20 | 1.00 | 0.04 |
| IL-22 | <0.01 | 1.00 | <0.01 | 0.78 | 1.00 | 0.84 |
| GM-CSF | 0.01 | 1.00 | <0.01 | 1.00 | 0.97 | 0.95 |
| TNFα | <0.01 | 1.00 | <0.01 | 1.00 | 1.00 | 1.00 |
| IFN-γ | 0.95 | 0.91 | 1.00 | 1.00 | 1.00 | 0.96 |
| CXCL2 positive reactions in IHC | <0.01 | <0.01 | 0.02 | 0.98 | 1.0 | 0.98 |
