# Supplementary material for: Inflammatory markers activation associated with vapor or smoke exposure in Wistar rats
Source: Front Immunol. 2025 Mar 21;16:1525166. doi: 10.3389/fimmu.2025.1525166 (PMC11968385; doi:10.3389/fimmu.2025.1525166)
Supplement: Supplementary file 4 [file Table2.docx]

## **Supplementary material**

Table II. Cytokines concentration (pg/ml) in groups

| Cytokine | Group mean±SE (95% CI) | | | | | |
| --- | --- | --- | --- | --- | --- | --- |
|  | 1 | 2 | 3 | 4 | 5 | 6 |
| IL-2 | 1234.70±80.03 (1074.91-1394.48) | 1623.09 ±80.03 (1463.31-1782.88) | 1196.22 ±80.03 (1036.43-1356.00) | 1253.08 ± 80.03 (1093.28-1412.86) | 1188.44 ± 80.03 (1028.65-1348.22) | 1311.25 ± 80.03 (1151.46-1471.04) |
| IL-4 | 737.27±136.07 (465.60-1008.96) | 1231.17±136.07 (959.48-1502.84) | 1112.19±136.07  (840.51-1383.86) | 856.33±136.07  (-268.37-1981.03) | 728.52±136.07 (456.84-1000.20) | 977.13±136.07  (705.45-1248.81) |
| IL-5 | 630.58±32.34 (566.01- 695.15) | 639.45±32.34 (574.88-704.01) | 616.26±32.34 (551.69-680.83) | 585.49± 32.34  (584.65-1128.02) | 568.61±32.34 (504.05-633.18) | 594.03±32.34 (529.47-658.60) |
| IL-6 | 1630.56±137.41  (1356.22-1904.91) | 2468.29±137.41  (2193.94-2742.63) | 1602.72±137.41  (1328.38-1877.06) | 1670.31±137.41  (1395.96-1944.65) | 1578.53±137.41  (1304.18-1852.87) | 1780.85±137.41  (1506.50-2055.19) |
| IL-9 | 2408.70±840.75  (730.07-4087.31) | 7530.50±840.75  (5851.88-9209.12) | 2441.93±840.75  (763.30- 4120.55) | 2526.15±840.75  (847.53- 4204.76) | 2221.15±840.75  (542.53- 3899.77) | 2792.01±840.75  (1113.39-4470.63) |
| IL-10 | 971.10±38.74  (893.76-1048.44) | 782.17±38.74  (704.83-859.51) | 792.73±38.74  (715.39-870.07) | 797.78±38.74  (720.45-875.13) | 776.77±38.74  (699.43-854.11) | 846.95±38.74  (769.61-924.30) |
| IL-13 | 1555.01±137.31 (1280.86-1829.16) | 2135.21±137.31  (1861.06-2409.36) | 1987.02±137.31  (1712.87-2261.17) | 1839.76±137.31  (1565.61-2113.92) | 1660.21±137.31  (1386.06-1934.37) | 2061.54±137.31  (1787.38-2335.69) |
| IL-17A | 2019.95±305.50  (1409.99-2629.89) | 2442.14±305.50  (1832.19-3052.09) | 1608.31±305.50  (998.36-2218.26) | 1339.31±305.50  (729.36-1949.26) | 1187.31±305.50  (577.36-1797.26) | 1581.25±305.50  (971.30-2191.21) |
| IL-17F | 1095.35±86.34  (922.96-1267.75) | 1318.44±86.34  (1146.05-1490.84) | 1131.10±86.34  (958.71-1303.49) | 1366.48±86.34  (1194.09-1538.88) | 1061.42±86.34  (889.02-1233.81) | 1513.29±86.34  (1340.90-1685.69) |
| IL-22 | 1718.36±114.94  (1488.87-1947.84) | 2299.68±114.94  (2070.19-2529.16) | 1689.96±114.94  (1460.48-1919.45) | 1834.31±114.94  (1604.82-2063.79) | 1620.96±114.94  (1391.48-1850.45) | 1813.93±114.94  (1584.44-2043.42) |
| GM-CSF | 812.33±140.26  (532.29- 1092.37) | 1440.08±140.26  (1160.04-1720.11) | 783.79±140.26  (503.75-1063.82) | 775.81±140.26  (495.77-1055.84) | 770.90±140.26  (490.87-1050.94) | 845.12±140.26  (565.08-1125.16) |
| TNFα | 897.30±99.37  (698.91-1095.69) | 1439.95±99.37  (1241.55-1638.34) | 864.80±99.37  (666.40-1063.19) | 876.48±99.37  (678.09-1074.87) | 839.09±99.37  (640.70-1037.48) | 866.65±99.37  (668.25-1065.04) |
| IFN-γ | 915.38±32.53  (850.43-980.32) | 956.23±32.53  (891.29-1021.18) | 962.90±32.53  (897.94-1027.84) | 886.63±32.53  (821.68-951.57) | 864.73±32.53  (799.78-929.67) | 903.19±32.53  (838.24-968.14) |
| CXCL2 (+) IHC reactions | 0.80±0.13 (0.55-1.04) | 2.56±0.12  (2.32-2.80) | 2.01±0.12  (1.76-2.25) | 2.08±0.12  (1.84-2.32) | 1.96±0.12  (1.72-2.20) | 2.08±0.12  (1.84-2.32) |
